# Electrical Detection of Pneumococcus through the Nanoparticle Decoration Method

**DOI:** 10.3390/s17092012

**Published:** 2017-09-02

**Authors:** Hannah Pyo, Cho Yeon Lee, Daehee Kim, Gyuhee Kim, Sangho Lee, Wan Soo Yun

**Affiliations:** 1 Department of Chemistry, Sungkyunkwan University (SKKU), Suwon 440-746, Korea; vyzkssk@gmail.com (H.P.); cylee04@skku.edu (C.Y.L.); nanokdh@gmail.com (D.K.); 2 Department of Biological Sciences, Sungkyunkwan University (SKKU), Suwon 440-746, Korea; khyhies77@naver.com (G.K.); sangholee@skku.edu (S.L.)

**Keywords:** pathogen detection, bacteria, microgap, biosensors, nanoparticle decoration

## Abstract

A simple method of nanoparticle decoration can be used in the detection of pneumococcus. After the pneumococcal bacteria were captured by an antibody (pneumococcal C-polysaccharide (PnC) antibody) between the interdigitated electrodes, the gold nanoparticles conjugated with the PnC antibodies were let to bind onto an outer membrane of the bacteria. Upon successfully dense decoration, the bacteria surface will become conductive owing to the metal nanoparticles, and a distinctive conductance change between the electrodes can be observed. Since this success ratio, or the probability of the conductance change, reflects the concentration of the analyte, a number of repeated measurements can be used in the quantification of the bacteria. In this way, we have successfully detected *S. pneumoniae* in the range of 10–10^8^ CFU/mL. The limit of detection in this work is lower than that in the commercial detection kit. We hope that the nanoparticle decoration method will play a role in the facile detection of various bacteria.

## 1. Introduction

The established method of bacteria analysis as standard protocols has been the bacterial culture and metabolic test [1]. For the detection of genetic material such as DNA or RNA, the polymerase chain reaction [2,3], and the ligase chain reaction [4] were also used to improve the accuracy of analysis. So far, alternative methods for the bacteria detection have been suggested with the use of various analytical techniques. As an optical technique, Arias et al. suggested the use of a fluorescence microscope and a UV/vis spectrophotometer to detect the fluorescent tag attached to the bacteria surface [5]. Joo et al. used antibody-immobilized TiO_2_ nanocrystals to enhance the surface plasmon resonance signal [6]. Quartz crystal microbalance, which monitors the resonance frequency change upon mass change, was also adopted in detecting the existence of bacteria [7,8].

In addition, a great amount of effort has been devoted to developing methods incorporating electric or electrochemical means, probably due to their advantageous characteristics in the implementation of practical sensors [9,10,11,12,13]. Some recent works include reports on the direct electric detection of pathogenic bacteria. Beck et al. claimed the real-time electrical detection of individual *Bacillus mycoides* was possible by dielectrophoretic manipulation of the cells [11]. Lu et al. showed that a direct electrical measurement of *E. coli* was possible when the experimental conditions are very carefully controlled [12]; recently, Mannoor et al. reported the electronic detection of *E. coli* based on antimicrobial peptide-functionalized microcapacitive electrode arrays [13]. The key observed value in these electrical methods is the change in electrical properties such as conductance and impedance, which can be greatly enhanced when the metal nanoparticles are incorporated in the detection scheme.

In this work, we used gold nanoparticles to decorate the surface of immobilized bacteria in the gap region of a microgap device, and the electrical conductance change upon the nanoparticle decoration was interpreted as the sign of the existence of bacteria. With this, the conductance change became greatly enhanced, and it sometimes reached 10^8^ times the initial value (before the nanoparticle decoration). However, the extent of the conductance change is quite random, showing a few orders of magnitudes in range. Therefore, although the conductance change indicates the existence of bacteria, the extent of the conductance change cannot reflect the concentration of the bacteria. To overcome this, in this work, we fabricated an integrated sensor chip with multiple microgap devices on it and used it to quantify the bacterial concentration. By counting the number of devices showing the conductance jump, we were able to quantify the *S. pneumoniae* at various concentrations.

## 2. Materials and Methods 

### 2.1. Materials

(3-Aminopropyl)trimethoxy-silane (APTMS), bovine serum albumin (BSA), phosphate-buffered saline (PBS), and thioglycolic acid (TGA) were purchased from Sigma-Aldrich (St. Louis, MO, USA). An anti-*S. pneumoniae* antibody (the pneumococcal C-polysaccharide (PnC) antibody) was purchased from Abcam (Cambridge, UK). BinaxNOW^®^ *Streptococcus pneumoniae* antigen card was purchased from Alere (Waltham, MA, USA). All chemicals and proteins were used without further purification. 

### 2.2. Instruments

A plasma processing system was used (Covance, FEMTO SCIENCE, Yongin-Si, Korea) for the hydroxylation of the SiO_2_ surface, and the surface was analyzed by Fourier transform infrared spectroscopy (FT-IR, VERTEX 70, Bruker Optics, Ettlingen, Germany) and atomic force microscopy (AFM, NX-10, Park systems, Suwon, Korea) after immobilization of the antibody. PnC antibody-conjugated gold nanoparticles were characterized with the use of dynamic light scattering (DLS, Nano-ZS90, Malvern, Worcestershire, UK) and ultraviolet-visible-near IR spectrophotometer (UV-VIS-NIR spectrophotometer, UV-3600, SHIMADZU, Kyoto, Japan). The size of gold nanoparticles were estimated from the images of scanning electron microscopy (SEM, JSM-7800, JEOL, Tokyo, Japan). The I-V characteristics were obtained using a probe station and a precision semiconductor parameter analyzer (4156A, Hewlett Packard, Palo Alto, CA, USA).

### 2.3. Functionalization of Microgap Devices

The microgap device composed of a pair of interdigitated electrodes (IDEs) were fabricated by photolithography. A sensor chip was designed to have 24 microgap devices on it. Before the functionalization of microgap devices, the devices were cleaned to eliminate any possible organic contaminants and were modified to have hydroxyl groups (-OH) on the SiO_2_ by O_2_ plasma for 10 min (100 W, 100 sccm, 1.5 Torr). The devices were immersed in a methanol solution containing 1% (v/v) APTMS for 1 h at room temperature and baked in an oven for 20 min at 70 °C. The IDEs made of gold were passivated with the use of 1 mM 1-dodecanethiol dissolved in absolute ethanol. Then, the devices were treated with a droplet of PnC antibody, 333.5 nM (50 µg/mL in 10 mM PBS buffer; pH 7.4), for 1 h at room temperature. As a final step, the antibody-immobilized devices were dipped into the PBS buffer containing the 1% (w/v) BSA solution for 30 min at room temperature.

### 2.4. Preparation of AuNP@PnC Antibody Probes

The spherical AuNPs were synthesized by a citrate reduction method [14]. Ten microliters of TGA was added to 1 mL of AuNP colloid (620 pM) and left overnight at room temperature for the ligand exchange from citrates to TGA on the AuNP surface. This mixture was centrifuged at 8000 rpm for 20 min, and, after the supernatant was removed, it was re-dispersed in 10 mM PBS buffer (pH 7.4). The PnC antibody (200 nM) in the PBS was added to the AuNP colloid (620 pM), and the mixture was then blended for about 2 h at room temperature. 

### 2.5. The Bacteria Detection through the Nanoparticle Decoration Method

For capturing the target bacteria on the gap region of the device, the sensor chip was immersed in *S. pneumoniae* solution in 10 mM PBS buffer for 1 h at room temperature, and the chip surface was gently washed by the PBS buffer and N_2_ blowing. The chip was then immersed into AuNP@PnC probe solution for 1 h at room temperature for the nanoparticle decoration on the bacteria surface. After gentle washing and drying, the electrical measurements were performed in a probe station equipped with a parameter analyzer. 

### 2.6. The Measurement of the On-Device Percentage (ODP) of the Sensor Chip

The ODP, the percent ratio of the number of on-devices (the devices showing the conductance jump) and the whole number of the devices tested, was obtained by counting the number of devices showing the conductance change more than 10 times the initial value or the noise level (about a few pS) [15]. The ODP values were then plotted against the concentration of *S. pneumoniae* to examine the correlation between them.

## 3. Results and Discussion

### 3.1. The Concept of Nanoparticle Decoration Method

**Figure 1:**
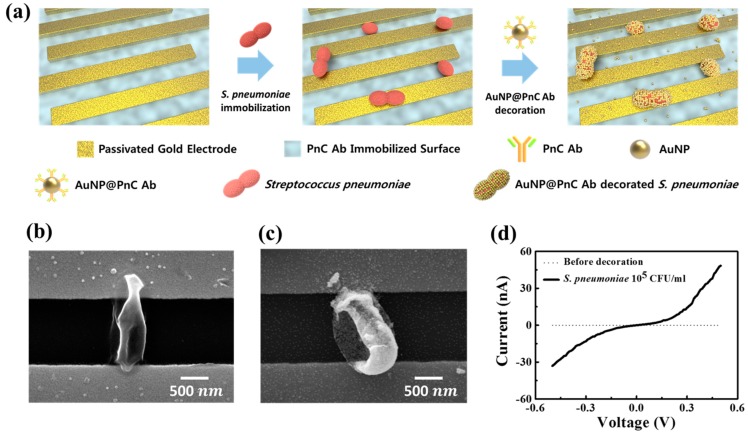
(**a**) The schematic diagram of the nanoparticle decoration method for the detection of *S. pneumonia*; (**b**) SEM images after immobilization of *S. pneumoniae* and (**c**) after nanoparticle decoration; (**d**) the I-V characteristics after cell immobilization (dotted line) and after AuNP@pneumococcal C-polysaccharide (PnC) antibody probes decoration on the cell surface (solid line).

Figure 1a depicts the process of the nanoparticle decoration method for the electrical detection of *S. pneumoniae*. The bacteria were selectively captured by the PnC antibody on the SiO_2_ surface between the electrodes so as to attain the probability of forming the electrical conduction bridge after the nanoparticle decoration. The surface of the *S. pneumoniae* was decorated by AuNP@PnC antibody probes, providing the electrical conduction pathway across the microgap. There can be some adhesion of probe particles on the device surface that is not mediated by the bacteria. However, these nanoparticles cannot give rise to the electric conduction between the electrodes because the size of the nanoparticle is very small (~25 nm) compared to the distance between the electrodes (~1.1 µm) and the surface density of the non-specifically adhering nanoparticle is not high enough to form a conduction channel. The device can become conductive only when the bacteria are decorated by the gold nanoparticles. 

Figure 1b,c is the SEM images of before-and-after decoration on the surface of the bacteria. As shown in the figures, the shape of the bacteria was puckered, probably due to the vacuum under which those images were taken (all samples were also dried for the observation). The bacteria surface became quite bright after the AuNP decoration due to the higher secondary electron current from the metal nanoparticles. When fully decorated, each bacteria surface can accept about a maximum of a few thousand AuNPs considering the size of AuNPs and bacteria. The amount (volume × concentration) of AuNPs used in the experiment was large enough to cover the surface of immobilized bacteria at higher concentrations. Figure 1d shows the I-V characteristics of the microgap device before and after the nanoparticle decoration. The conductance change was about 10^6^-fold in this case, which is large enough to unambiguously distinguish the change.

### 3.2. The Structure of the IDE Device and the Surface Functionalization of Silicon Oxide Surface

**Figure 2:**
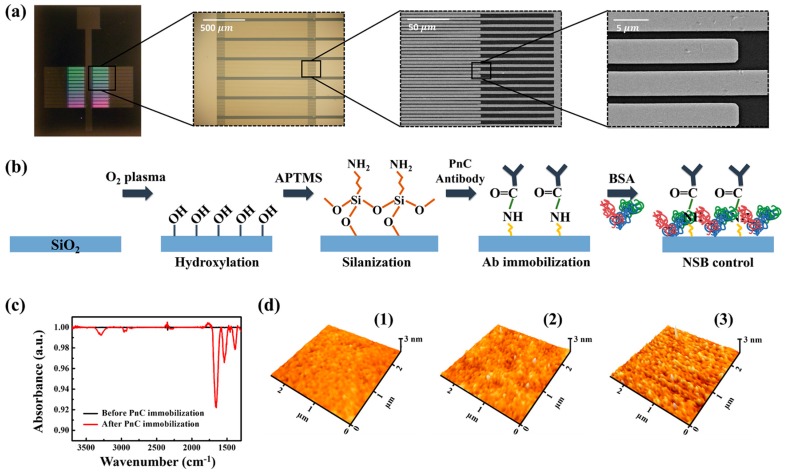
(**a**) The images of an integrated gap device having 24 microgap devices. Zoomed-in images show detailed structure of IDE device. (**b**) Chemical process for the functionalization of silicon oxide surface. (**c**) ATR-IR spectrum before and after the PnC immobilization. (**d**) AFM images of the silicon oxide region of the microgap device: (**d1**) Before silanzation; (**d2**) after silanization; (**d3**) after immobilization of the PnC antibody.

To improve the bacteria capturing efficiency, the gap sensor was designed to have IDE structure, where the total length of the 1.1 µm gap is about 4 × 10^4^ µm and the optical and electron microscope images are shown in Figure 2a. Since the major axis length of *S. pneumoniae* is from about 1–1.5 µm (single cell) to 2–3 µm (diplococci), the gap distance was set to be 1.1 ± 0.1 µm, which is comparable to the size of the bacteria.

Figure 2b shows a schematic illustration of the surface chemical reaction process on the gap resion. Following plasma oxidation on the SiO_2_ surface, a series of chemical processes was adopted to immobilize the capturing antibody PnC on that surface. BSA was used for the back-filling of the amine-activated surface to reduce the chance of non-specific binding of gold nanoparticles as well as interfering contaminants. Figure 2c,d shows ATR-IR spectrum and AFM topography images taken from the SiO_2_ surface following the chemical treatment steps. The ATR-IR spectrum obtained after the PnC immobilization exhibited multiple peaks of N–H bending at ~1560 cm*^−^*^1^, C=O stretching at ~1660 cm*^−^*^1^, and N–H stretching at ~3290 cm*^−^*^1^, implying that the PnC immobilization was successful. AFM analysis was adopted to examine the change in surface morphology of the SiO_2_ region of the device accompanied with the antibody immobilization process. As shown in Figure 2d, the surface was getting rougher as the process proceeded, indirectly supporting the successful immobilization of the PnC. The root-mean-sqaure roughness (R_q_) values of the initial, silinaized, and PnC-immobilized SiO_2_ surface were estimated to be about 0.099 nm, 0.171 nm, and 0.207 nm, respectively.

### 3.3. AuNP@PnC Antibody Probes

**Figure 3:**
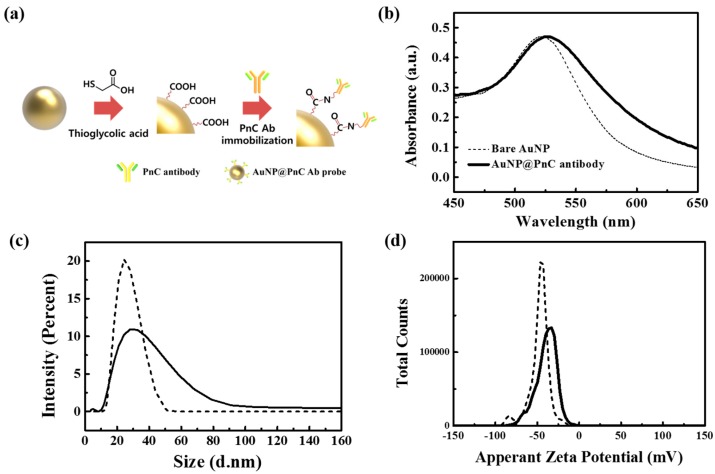
(**a**) The schematic illustration of antibody immobilization process on the AuNP surface. The analysis of the bare AuNPs and PnC-attached AuNP probes: (**b**) UV-VIS spectra, (**c**) DLS data, and (**d**) zeta potential data (dotted line: bare AuNPs, solid line: AuNP@PnC probes).

For the decoration on the bacteria surface, gold nanoparticles of 25 ± 4 nm in diameter were prepared and PnC antibodies were attached on the nanoparticle surface via a linker molecule, TGA. The procedure and the analysis data with UV/vis and DLS systems were shown in Figure 3. Upon antibody immobilization all the UV/vis and the DLS data showed a measurable change. UV/vis peak was shifted from ~520 nm to ~525 nm, and the average hydrodynamic radius estimated from DLS measurements was changed from ~20 to ~37 nm. In addition, the surface zeta potential was changed from about −47 mV to about −39 mV. Since the PnC antibody alone showed a lower value (−10 mV) in zeta potential than did the AuNP, the measured value of the nanoparticle probes also indirectly supports the formation of the AuNP@PnC probes. 

### 3.4. Detection of AuNP@PnC Antibody Decorated S. pneumoniae

As shown in Figure 1d, upon successful bridging of the microgap with the nanoparticle-decorated bacteria, the conductance of the device was substantially enhanced. However, it was not always successful. The bacteria sometimes could not be located on the electrode surface, and the bridging was sometimes incomplete. In addition, even though the bacteria were immobilized at the right position, the nanoparticle decoration was sometimes sparse (Figure S1). Therefore, the conductance jump was not always observed, even in cases where there was a high concentration of bacteria, meaning that the existence of the bacteria could not be determined, or that the measurement of their concentration could not be performed, with the use of a single device.

**Figure 4:**
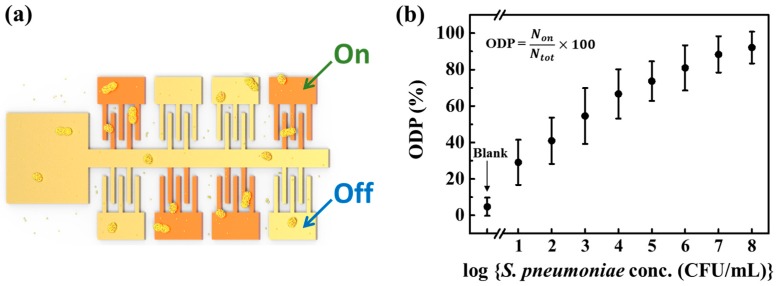
(**a**) The conceptual representation of the detection of bacteria with the use of integrated microgap devices through the nanoparticle decoration method. (**b**) The ODP curve with respect to *S. pneumoniae* concentration. Error bars represent the 95% confidence intervals.

Interestingly, however, the success ratio, or the ratio between the number of devices showing the conductance jump and the whole number of devices tested, was found to be closely related to the concentration of the bacteria. Figure 4 shows the concept of this approach and the result obtained. Here, on-device-percentage (ODP) is the success ratio: the percentage of on-devices (the devices showing the conductance jump). As one can see in the figures, the ODP value increased as the concentration of *S. pneumoniae* was increased. The range of quantification spans from 10 to 10^8^ CFU/mL. The ODP value at the lowest concentration of 10 CFU/mL was about 28%, which is well above the blank (the sensor chip immersed into AuNP@PnC probe solution only without the *S. pneumoniae*) value of about 5%. Therefore, we can say that this method of combing the microgap sensor chip and nanoparticle decoration is useful at this low concentration level, particularly when compared with the results obtained with the commercial detection kit where *S. pneumoniae* of 10^6^–10^7^ CFU/mL was hardly discernable (Figure S2). It seems that the error bars in Figure 4b need to be improved. The error in the detection of *S. pneumoniae* by the nanoparticle decoration method may arise mainly from the randomness of the location of bacterial cells on the device surface and the incompleteness of the nanoparticle decoration. In order to alleviate those effects, one may consider the increase in the number of devices or the reduction of the gap distance, which should be verified by further study. 

### 3.5. Selectivity Test of S. pnuemoniae Detection through the Nanoparticlce Decoration Method

**Figure 5:**
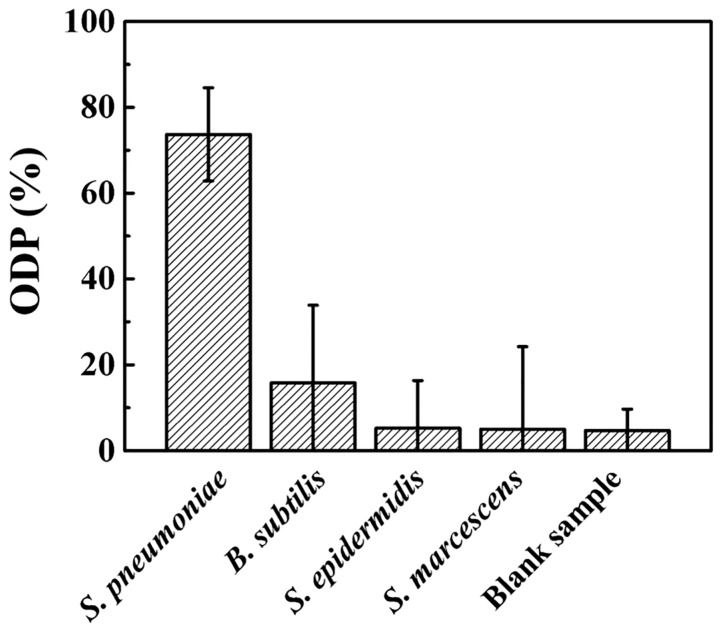
The selectivity of the nanoparticle decoration method using the PnC antibody. Error bars represent the 95% confidence intervals.

To examine the selectivity of the proposed electrical detection method, the aforesaid process was performed on both specific and non-specific target bacteria. Figure 5 shows the comparison of the ODP values for samples containing 1.0 × 10^5^ CFU/mL of *Bacillus subtilis* (*B. Subtilis*), *Staphylococcus epidermidis* (*S. epidermidis*), *Serratia marcescens* (*S. marcescens*) with that of *S. pneumoniae*. As shown in the figure, the ODP values from the non-specific bacteria were negligible compared to that of the specific target bacteria. The small values of ODP values in those non-specific cases were attributable to the presence of aggregated nanopaticles on the device surface. Although the individual particle size is small enough not to form the conduction pathway from just the random non-specific adhesion, occasional immobilization of the particle aggregates exceeding the gap distance will lead the conductance jump (Figure S3). As can be seen from the error bars in Figure 5, this nanoparticle aggregation seemed to be slightly more promoted in the sample solutions than in the blank solution.

## 4. Conclusions

In summary, we suggest that the simple electrical detection of *S. pneumoniae* is possible through the nanoparticle decoration method. The electrical conduction of a microgap device changed substantially upon the successful formation of the conduction pathway. Since the success ratio of the bridging of the electrode gap correlated well with the bacteria concentration, the ODP value obtained from different target concentrations is a good parameter for the bacteria concentration in the sample solutions. The minimum measurable concentration of the *S. pneumoniae* reached about 10 CFU/mL, which is a few orders of magnitude lower than that of the commercial strip kits. In this work, the PnC antibody was used for the selective detection of *S. pneumoniae*. Therefore, incorporating this method in the detection of different pathogenic bacteria will be quite straightforward since we only need to adopt a different antibody for the different target.

## Supplementary Materials

The following are available online at www.mdpi.com/1424-8220/17/9/2012/s1. Figure S1: Appearance of bacteria with different degree of decoration. In this case, the time for the immersion in the AuNP@PnC probe solution is different: (a) 30 min and (b) 1 h; Figure S2: Detection of *S. pneumoniae* R6 using commercially available strip kits (Alere BinaxNOW *S. pneumoniae* antigen card); Figure S3: SEM image of aggregated AuNP@PnC probes bridging the two electrodes.
