# Supplementary material for: Electrical Detection of Pneumococcus through the Nanoparticle Decoration Method
Source: Sensors (Basel). 2017 Sep 2;17(9):2012. doi: 10.3390/s17092012 (PMC5621115; doi:10.3390/s17092012)
Supplement: Supplementary file 1 [file sensors-17-02012-s001.pdf]

Supplementary Materials: Electrical Detection of Pneumococcus through the Nanoparticle Decoration Method

Hannah Pyo[1], Cho Yeon Lee[1], Daehee Kim[1], Gyuhee Kim[2], Sangho Lee[2], and Wan Soo Yun[1,*]

[1] Department of Chemistry, Sungkyunkwan University (SKKU), Suwon 440-746, Korea; vyzkssk@gmail.com (H.P.); cylee04@skku.edu (C.Y.L.); nanokdh@gmail.com (D.K.)
[2] Department of Biological Science, Sungkyunkwan University (SKKU), Suwon 440-746, Korea; khyhies77@naver.com (G.K.); sangholee@skku.edu (S.L.)
* Correspondence: wsyun87@skku.edu; Tel.: +82-(31)-290-7075

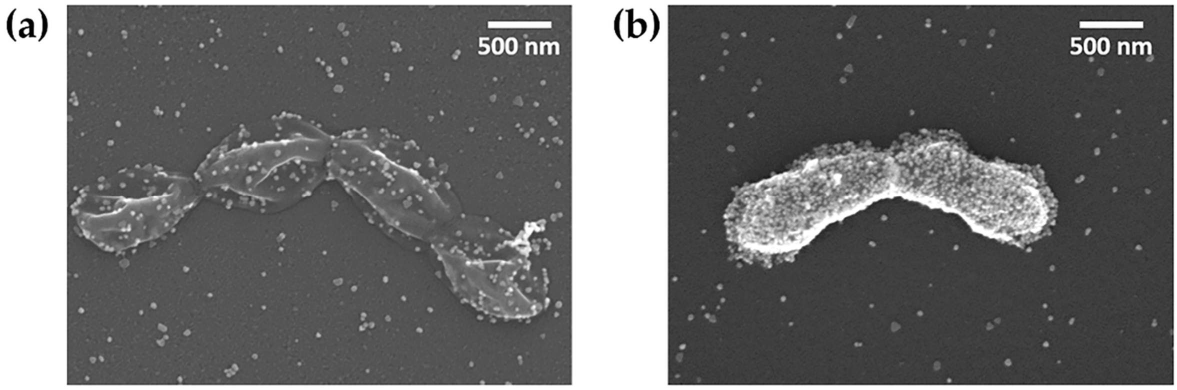

Figure S1. Appearance of bacteria with different degrees of decoration. In this case, the time for the immersion in the AuNP@PnC probe solution is different: (a) 30 min and (b) 1 h.

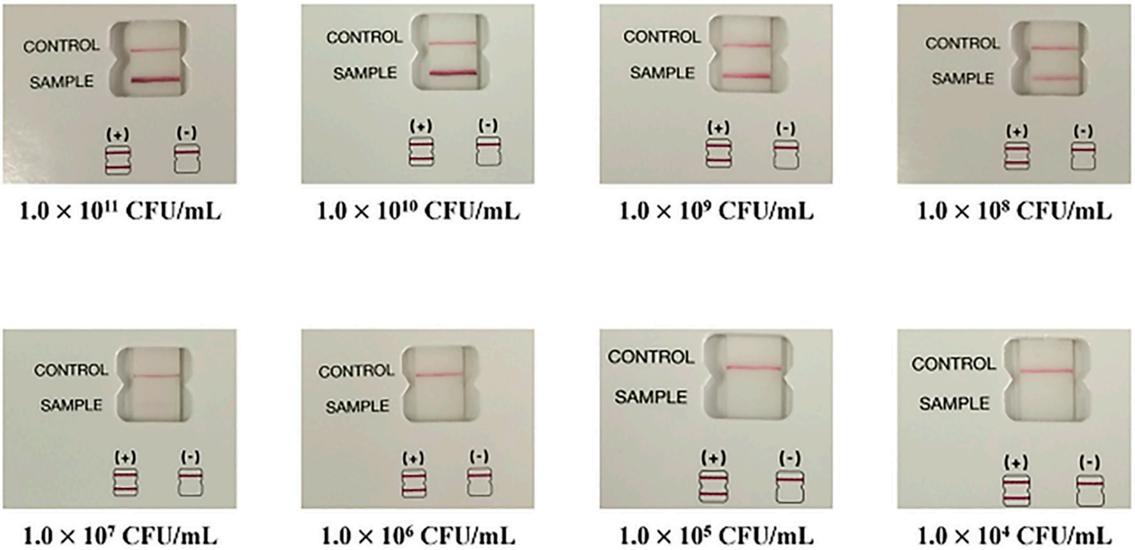

Figure S2. Detection of *S. pneumoniae* R6 using commercially available strip kits (Alere BinaxNOW *S. pneumoniae* antigen card).

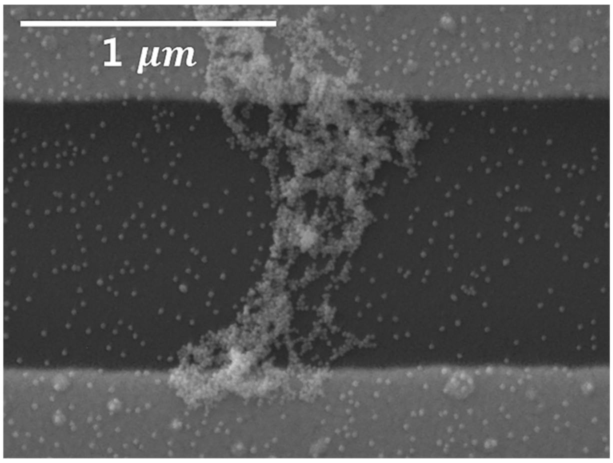

Figure S3. SEM image of aggregated AuNP@PnC probes bridging the two electrodes.
